# Antibiofilm activity of three-dimensional bacterial nanocellulose scaffolds containing antimicrobial agents

**DOI:** 10.1590/0103-644020256820

**Published:** 2026-01-30

**Authors:** Fernanda Keil Kressin, Isabela Aparecida Stahelin, Gilmar da Rosa Souza, Karina Cesca, Ricardo Ruiz Mazzon, Rayssa Sabino-Silva, Taynara Santos Goulart, Cleonice da Silveira Teixeira, Lucas da Fonseca Roberti Garcia, Josiane de Almeida

**Affiliations:** 1 Department of Dentistry, Endodontics Division, University of Southern Santa Catarina, Palhoça, Santa Catarina, Brazil; 2 Department of Chemical Engineering, Federal University of Santa Catarina, Florianópolis, Santa Catarina, Brazil; 2 Department of Microbiology, Immunology and Parasitology, Federal University of Santa Catarina, Florianópolis, Santa Catarina, Brazil; 2 Department of Dentistry, Endodontics Division, Health Sciences Center, Federal University of Santa Catarina, Florianópolis, Santa Catarina, Brazil

**Keywords:** Bacterial Nanocellulose, Multispecies Biofilm, Regenerative Endodontics

## Abstract

Studies have focused on the use of alternative scaffolds for Regenerative Endodontic Procedures (REPs) because current materials still pose significant technical limitations. The purpose of this *in vitro* study was to evaluate the antibiofilm activity of three-dimensional bacterial nanocellulose (BNC) scaffolds incorporated with 0.12% chlorhexidine digluconate (BNC/CHX), 1% amoxicillin (BNC/AMOX), and 1% clindamycin (BNC/CLI). BNC scaffolds without an antimicrobial agent composed the control group (BNC/C). BNC scaffolds were immersed in a multispecies culture of *E. faecalis*, *A. naeslundii*, and *S. sanguinis*, which served as a substrate for biofilm growth. After 24 hours, 7, and 15 days, the mean value of colony-forming units (CFUs) was determined. Multispecies biofilm formation on the BNC scaffolds was assessed under Scanning Electron Microscopy (SEM) and Confocal Laser Scanning Microscopy (CLSM). For the biofilm cell viability test, the mean value of CFU/mL for each sample was determined, and the data were normalized by taking the base-10 logarithm (log^10^) of each CFU/mL value. Data were analyzed by Two-way ANOVA, followed by Bonferroni’s post-hoc multiple comparisons (α=5%). In the 24h period, BNC/AMOX demonstrated significantly lower CFUs mean values than BNC/CHX, BNC/CLI, and BNC/C (p<0.05). CFUs mean values increased throughout the experimental periods, with no significant difference compared to the other groups (p>0.05). SEM and CLSM analysis revealed, in the initial periods, a lower number of microorganisms in the experimental groups compared to the control group. BNC scaffolds containing 1% amoxicillin and 1% clindamycin demonstrated greater antimicrobial activity against a multispecies biofilm within the initial periods of incubation

## Resumo

Estudos têm investigado o uso de *scaffolds* alternativos para Procedimentos Endodônticos Regenerativos (PERs), uma vez que ainda existem limitações técnicas significativas nos materiais atualmente empregados. O objetivo deste estudo *in vitro* foi avaliar a atividade antibiofilme de *scaffolds* tridimensionais de nanocelulose bacteriana (BNC) incorporados com 0,12% de digluconato de clorexidina (BNC/CHX), 1% de amoxicilina (BNC/AMOX) e 1% de clindamicina (BNC/CLI). *Scaffolds* de BNC sem agente antimicrobiano compuseram o grupo controle (BNC/C). Os *scaffolds* de BNC foram imersos em uma cultura multiespécies de *E. faecalis*, *A. naeslundii* e *S. sanguinis*, servindo como substrato para o crescimento do biofilme. Após 24 horas, 7 e 15 dias, determinou-se o valor médio de unidades formadoras de colônia (UFCs). A formação de biofilme multiespécies nos *scaffolds* de BNC foi avaliada por Microscopia Eletrônica de Varredura (MEV) e por Microscopia Confocal de Varredura a Laser (CLSM). Para o teste de viabilidade celular do biofilme, o valor médio de UFC/mL de cada amostra foi determinado e os dados foram normalizados pela transformação logaritmo de base 10 (log^₁₀^) de cada valor de UFC/mL. Os dados foram analisados pelos testes de ANOVA de dois fatores, seguida do teste de comparações múltiplas post-hoc de Bonferroni (α=5%). No período de 24h, o BNC/AMOX apresentou valores médios de UFCs significativamente menores do que BNC/CHX, BNC/CLI e BNC/C (p<0,05). Os valores médios de UFCs aumentaram ao longo dos períodos experimentais, sem diferença significativa em relação aos demais grupos (p>0,05). A análise por MEV e CLSM revelou, nos períodos iniciais, um número menor de microrganismos nos grupos experimentais em comparação ao grupo controle. Scaffolds de BNC contendo 1% de amoxicilina e 1% de clindamicina demonstraram maior atividade antimicrobiana contra o biofilme multiespécies nos períodos iniciais de incubação.

**Figure:**
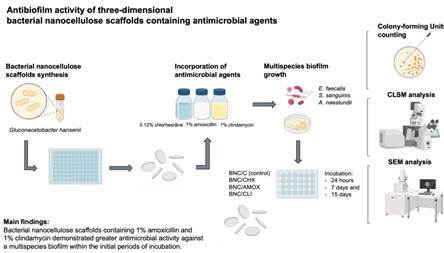

## Introduction

Regenerative Endodontic Procedures (REPs) are biologically-based clinical therapies designed to induce the development of the root structure as well as cells within the dentin-pulp complex ^(^ ^1^. However, to achieve pulp tissue regeneration and restore physiological pulp functions, specific challenges must be overcome ^(^ ^2^.

The success of REPs depends on a reduced number of microorganisms within the root canal system ^(^ ^1^. Considering that mechanical instrumentation cannot be performed due to the presence of thin and fragile root canal walls, the disinfection occurs almost exclusively through chemical preparation ^(^ ^2^. Up to this point, the root canal system disinfection has been advocated through irrigation with 2.5% NaOCl solution and the use of intracanal dressing ^(^ ^2^. The intracanal dressing used can be either a calcium hydroxide paste or a triple antibiotic paste (TAP) (metronidazole, ciprofloxacin, and minocycline) ^(^ ^1^. Despite being highly recommended by the American Academy of Endodontists ^(^ ^1^, these antimicrobial agents have toxic effects on stem cells ^(^ ^3^ ^,^ ^4^. Additionally, even after proper chemical preparation, bacterial biofilms may persist within the root canal system ^(^ ^5^.

After disinfection, the root canal remains empty, requiring the use of a scaffold ^(^ ^6^. Such a procedure is another challenge in the regeneration process ^(^ ^6^. Scaffolds are three-dimensional, biomimetic structures designed to provide a temporary extracellular matrix that supports and guides the biological processes required for tissue regeneration ^(^ ^7^. They serve not only as a physical framework for the migration, adhesion, proliferation, and differentiation of stem cells, but also as a bioactive environment capable of modulating cellular behavior and promoting signaling cascades essential for tissue repair ^(^ ^7^. An ideal scaffold must possess adequate porosity and interconnectivity to enable cell colonization, nutrient diffusion, and waste removal, while also permitting angiogenesis and revascularization, both critical for pulp vitality and sustained tissue development ^(^ ^7^ ^,^ ^8^. In REPs, scaffolds play a pivotal role in orchestrating the deposition of new dentin, thickening the radicular walls, and ultimately allowing for the completion of root maturation ^(^ ^7^ ^,^ ^8^ ^,^ ^9^. Moreover, beyond their structural support, scaffolds can be functionalized with bioactive molecules, growth factors, or antimicrobial agents, thereby enhancing their regenerative and protective potential within the root canal environment ^(^ ^7^ ^,^ ^8^ ^,^ ^9^.

Currently, the majority of REPs are based on the use of endogenous structures, such as blood clot, platelet-rich plasma, and platelet-rich fibrin ^(^ ^8^. However, significant technical limitations are observed, such as the difficulty in forming an intracanal blood clot after inducing bleeding or performing venipuncture to obtain plasma and fibrin ^(^ ^8^. Thus, research advances have focused on the use of alternative scaffolds ^(^ ^8^ ^,^ ^9^. Bacterial nanocellulose (BNC) is a natural three-dimensional cellulose biosynthesized by the bacterium *Gluconacetobacter hansenii* ^(^ ^10^ ^,^ ^11^. It is widely employed in tissue engineering ^(^ ^10^ ^,^ ^11^ ^,^ ^12^ ^,^ ^13^ due to its biocompatibility, flexibility, purity, and mechanical stability ^(^ ^14^. Furthermore, BNC allows for the immobilization of active molecules within its nanofiber network ^(^ ^15^ and possesses a slow drug release system ^(^ ^10^. Kichler et al. ^(^ ^15^ have reported the cytocompatibility of BNC membranes in fibroblasts of the periodontal ligament.

Among antimicrobial agents, chlorhexidine digluconate has been extensively used in Endodontics due to its broad-spectrum activity and substantivity, even at low concentrations such as 0.12%, which reduces cytotoxicity while maintaining antibacterial effectiveness ^(^ ^16^. Amoxicillin is a widely prescribed β-lactam antibiotic with activity against *Enterococcus faecalis* and other resistant species frequently associated with endodontic infections ^(^ ^17^. Clindamycin, a lincosamide antibiotic, has been indicated as an alternative in cases of penicillin allergy and is active against anaerobic bacteria prevalent in root canal infections ^(^ ^18^.

Bearing this in mind, the incorporation of different antimicrobial agents onto the nanofiber network of BNC with slow and prolonged release might be an innovative disinfection approach with significant potential for use in REPs. Therefore, this *in vitro* study aimed to evaluate the antibiofilm activity of three-dimensional BNC scaffolds impregnated with 0.12% chlorhexidine digluconate (BNC/CHX), 1% amoxicillin (BNC/AMOX), and 1% clindamycin (BNC/CLI). Different evaluation periods of 24 hours, 7 days, and 15 days were chosen to provide a comprehensive assessment of both immediate and prolonged antibiofilm activity. These time points represent (i) the short-term effect of antimicrobials (24 h), (ii) an intermediate phase compatible with early clinical outcomes (7 days), and (iii) an extended release profile (15 days), which is critical for preventing bacterial regrowth in the root canal system ^(^ ^19^. The following null hypotheses were tested: (i) there would be no difference in the antibiofilm activity among the different BNC scaffolds (BNC, BNC/CHX, BNC/AMOX, and BNC/CLI); and (ii) there would be no difference in the antibiofilm activity of each scaffold across the incubation periods (24 hours, 7 days, and 15 days).

## Materials and methods

### BNC scaffolds synthesis and purification

One hundred and twenty BNC scaffolds were produced using an inoculum of *Gluconacetobacter hansenii* bacteria (ATCC 23769) obtained from the Collection of Tropical Culture (CCT, André Tosello Foundation, Campinas, SP, Brazil). The inoculum was prepared three days before the start of the experiments. Cultures of the bacteria were suspended in 1 mL of mannitol-based growth medium (25 g/L mannitol, 5.0 g/L yeast extract, and 3.0 g/L bacteriological peptone diluted in distilled water). The suspension was vortexed for 60 seconds and subsequently allowed to rest for 10 seconds. Following this, the optical density was adjusted to OD_660_ = 0.150, after which a 1:10 dilution was prepared using the same culture medium. The inoculum was then dispensed into 96-well culture plates and incubated under static conditions at 26°C for seven days. Under these conditions, *Gluconacetobacter hansenii* synthesizes a continuous nanocellulose pellicle at the air-liquid interface, where oxygen availability is highest. This pellicle gradually thickened over the incubation period, forming uniform BNC membranes characteristic of static fermentation. At the end of seven days, the pellicles were carefully lifted from the air-liquid interface using sterile forceps to avoid disrupting the nanofiber structure. Excess medium was allowed to drain, and the pellicles were standardized into individual scaffold discs measuring 6 mm in diameter and 1 mm in thickness. Scaffolds were then collected and purified in 0.1 M sodium hydroxide at 50°C for 24 hours to remove all bacterial cells and remnants of the culture medium retained within the nanofiber network. The BNC scaffolds were repeatedly rinsed with distilled water until the washing solution reached a neutral pH equivalent (7.0) to that of the distilled water used. Finally, the BNC scaffolds were sterilized through autoclaving for 20 minutes at 121°C ^(^ ^15^.

### Oxidation and incorporation of antimicrobial agents into BNC scaffolds

The oxidation process of the nanofibers was carried out to make them chemically more susceptible to the incorporation of antimicrobial agents. The BNC scaffolds were treated by immersion in a nitric acid and phosphoric acid mixture at a 2:1 (v/v) ratio. Subsequently, sodium nitrite was added at a concentration of 7% (m/v). The reaction was carried out for 24 hours under agitation, at 25 °C, and protected from light. After completion, the scaffolds were transferred to an aqueous glycerol solution (0.2% w/w) for 15 minutes in order to neutralize residual oxidizing agents. The scaffolds were then rinsed with acetone and left to dry at room temperature (25°C). Subsequently, the scaffolds were placed in an aqueous solution containing 0.2% (w/w) glycerol for 15 minutes to neutralize residual oxidizing agents. They were then rinsed with acetone and dried at 25 °C under ambient conditions. The antimicrobial agents selected for this study were prepared in their respective pharmaceutical forms: 0.12% chlorhexidine digluconate was used as an aqueous solution (Rio Química, São José do Rio Preto, SP, Brazil), while 1% clindamycin (Drogaria Minas Brasil, Belo Horizonte, MG, Brazil) and 1% amoxicillin (Magistrale Farmácia, Florianópolis, SC, Brazil) were obtained in powdered form and dissolved in distilled water immediately before use to ensure complete solubilization and stability. The resulting solutions were incorporated into the oxidized BNC scaffolds through passive diffusion under constant stirring. The scaffolds were immersed in the respective antimicrobial solutions for 24 hours at room temperature to ensure adequate interaction between the nanofiber network and the active molecules ^(^ ^15^. BNC scaffolds containing the antimicrobial agents were stored in sterile airtight polypropylene containers, protected from light, at room temperature (25 °C) for a maximum of 48 hours prior to experimental use, in order to preserve the stability of the incorporated compounds.

### Experimental and control groups

Scaffolds were randomly assigned to four groups (n = 30 each), as follows: BNC/C (Control group - negative control) - oxidized BNC scaffolds without antimicrobial agents; BNC/CHX - BNC with 0.12% chlorhexidine digluconate; BNC/CLI - BNC with 1% clindamycin; and BNC/AMOX - BNC with 1% amoxicillin.

The BNC/C group served as the negative control for all assays, undergoing the same synthesis, purification, oxidation, and sterilization protocols as the experimental scaffolds. This ensured that any residual antimicrobial effect or microbial contamination was eliminated, allowing accurate assessment of the incorporated agents’ intrinsic antibiofilm activity ^(^ ^15^.

### Multispecies biofilm growth

Three facultative anaerobic bacterial species commonly found in cases of pulp necrosis in teeth with incomplete root formation were utilized ^(^ ^19^: *Enterococcus faecalis* (ATCC 29212), *Streptococcus sanguinis* (ATCC 10556), and *Actinomyces naeslundii* (ATCC 43146). Each species was initially grown separately by overnight incubation of 500 μL in 10 mL of brain-heart infusion broth (BHI) (Kasvi, Curitiba, PR, Brazil; pH 7.1) at 37°C under aerobic conditions ^(^ ^15^. Following incubation, the cultures were individually adjusted to the same optical density (OD ≈ 0.5), which corresponds to a standardized and reproducible bacterial load commonly used in biofilm studies. Although individual CFU counts were not performed for each species prior to inoculum preparation, the OD-based adjustment ensured equivalent cell densities before the cultures were pooled in equal proportions (1:1:1) to generate the multispecies suspension. This mixed bacterial suspension was subsequently diluted 1:100 in fresh BHI medium and used to inoculate the 24-well plates, which were filled with 1.5 mL/well of growth medium. Standardized BNC scaffolds (Ø = 5 mm) from each experimental group were then placed in the wells, serving as substrates for multispecies biofilm development. The plates containing the inoculum and scaffolds were sealed and incubated under aerobic conditions at 37°C for 24 hours, 7 days, and 15 days.

### Biofilm sampling and Colony-forming Units counting

After the experimental periods of incubation (24 hours, 7 days, and 15 days), the BNC scaffolds removed from the wells were washed with phosphate-buffered saline (PBS) (Êxodo Científica, Sumaré, SP, Brazil) to remove non-adherent biofilm cells. For determining the antibiofilm activity effect (n=6), the samples were transferred to plastic containers with 2 mL of PBS and sonicated for 15 minutes at an amplitude of 40 W to remove any remaining biofilms adhered to the scaffolds. The resulting suspensions were serially diluted (10_1_, 10_2_, 10_3_, 10_4_, and 10_5_), and 10μL aliquots were plated in triplicate on BHI agar plates. The plates were then incubated aerobically at 37ºC for 24 to 48 hours. After incubation, the plates corresponding to dilutions yielding 30-300 colonies were selected for quantification. The colonies were counted manually, and the number of CFUs/mL was calculated according to standard microbiological criteria: CFU/mL = (number of colonies × dilution factor) / plated volume (0.01 mL). The mean CFU/mL value for each sample was obtained from triplicate plating.

### Scanning Electron Microscopy (SEM) analysis

For the qualitative assessment of multispecies biofilm formation, two scaffolds from each experimental and negative control group of each period (24 hours, 7 days, and 15 days) were observed under SEM (JSM-6390 LV; JEOL Ltd., Tokyo, Japan) (n = 2). Initially, the scaffolds were fixed in 2.5% glutaraldehyde buffered with 0.2 M cacodylate for 12 hours at 4°C. Subsequently, they were washed with cacodylate buffer for 1 hour, dehydrated with increasing alcohol gradients (25%, 50%, 75%, and 95% for 20 minutes each, and 100% for 1 hour), mounted on stubs, and sputter-coated with a gold-palladium thin layer (300 Å). The images were obtained with the SEM operating at 10 kV. The samples were examined under magnifications ranging from 1000× to 6,000×.

### Confocal Laser Scanning Microscopy (CLSM) analysis

The presence of viable and non-viable microorganisms adhered to the BNC scaffolds was qualitatively determined through CLSM (Olympus Europa Holding GmbH, Hamburg, Germany) (n=2). The BNC scaffolds were aseptically removed from the 24-well plates after each experimental period (24 hours, 7 days, and 15 days). Subsequently, they were placed on a glass base (20 mm in diameter and 0.17 mm thick) and stained with the "Live/Dead" kit (BacLight_TM_ L-13152; Molecular Probes, Inc., Eugene, OR, USA). The multispecies biofilm was stained with the SYTO 9 and propidium iodide dyes, which were applied to the samples for 20 minutes at a 1:1 ratio (total volume = 50 μL), kept protected from light. The microscope was set at 473 nm laser emission for SYTO 9 and 559 nm emission for propidium iodide. The samples were observed using a 50× oil-immersion objective lens with a numerical aperture of 1.4, and the confocal pinhole was adjusted to a diameter of 30 mm. The fluorescence of the stained microorganisms was visualized, and the images were taken under magnifications ranging from 200× to 1000×. Microorganisms stained in green (SYTO 9) indicated viable microbial cells, while those stained in red (propidium iodide) indicated non-viable microbial cells. The analysis aimed to characterize the general distribution of viable (green) and non-viable (red) microorganisms on the scaffold surface; therefore, no quantitative live/dead cell counting was performed.

Both SEM and CLSM analyses were conducted by a trained examiner in a blinded fashion. The images utilized for calibration were excluded from the subsequent assessment. Image assessment was repeated after a one-month interval using digitally archived SEM and CLSM micrographs, ensuring that no physical storage of the samples was required. This procedure was adopted to minimize examiner bias and to allow reproducible re-evaluation under identical digital imaging conditions. The intra-examiner reliability was deemed excellent (Kappa = 0.80).

### Statistical analysis

The homogeneity of variances was determined using the Levene test, and the normality of residuals was determined using the Shapiro-Wilk test. For the biofilm cell viability test, the mean CFU/mL for each sample per period was determined, and the data were normalized by taking the base-10 logarithm (log_10_) of each CFU/mL value. The effects of the different experimental scaffolds on biofilm cell viability and the periods of incubation were analyzed by the Two-way ANOVA, followed by Bonferroni’s post-hoc multiple comparisons. The significance level was set at 5%. All statistical tests were conducted using the SPSS software version 21.0 (IBM, Armonk, NY, USA). Descriptive methods were used to analyze SEM and CLSM images. The analysis was conducted by a trained examiner in a blinded fashion. The images utilized for calibration were excluded from the subsequent assessment. Image assessment was repeated after a one-month interval to mitigate potential bias. The intra-examiner reliability was deemed excellent.

## Results

### Colony-forming Units counting

The mean values (log^10^) of CFUs/mL, corresponding to the remaining viable bacteria adhered to BNC scaffolds at 24 hours, 7 days, and 15 days, are displayed in Table 1.

BNC/C exhibited a progressive increase in the viable bacterial mean values across the different incubation periods. However, a significant difference in the amount of viable microorganisms was observed in the control group only at 15 days of incubation in comparison with the initial period (p<0.05).

BNC/CHX presented viable bacterial mean values similar to BNC/C across all incubation periods (p>.05). BNC/CHX had significantly lower values of microorganisms adhered to the scaffolds at the 24-hour period compared to 7 and 15 days (p<0.05). In the 7-day period, there was a significant increase in adherent microorganisms, which remained similar to the 15-day incubation period (p>0.05).

BNC/CLI had a lower amount of adhered microorganisms in the initial 24 hours when compared to the 15-day period (p<0.05). Additionally, significantly lower amount of microorganisms was observed in the 24-hour and 7-day periods when compared to BNC/CHX and BNC/C (p<0.05). The adherence of microorganisms to the scaffolds was similar to BNC/CHX and BNC/C in the 15-day period (p>0.05).

Similar to BNC/CLI, in the 24-hour period, BNC/AMOX showed significantly lower mean values of adhered microorganisms compared to the 15-day period (p<0.05). Furthermore, in the initial 24 hours, BNC/AMOX demonstrated significantly lower CFU mean values than BNC/CHX, BNC/CLI, and BNC/C (p<0.05). However, in the 15-day period, the values were similar to the other groups (p>0.05).

**Table 1:** Mean values (log^10^) and standard deviation (SD) of CFUs/mL corresponding to viable bacteria adhered to the BCN scaffolds incorporated with the different antimicrobial agents at 24 hours, 7 days, and 15 days.

Group	Log CFUs/mL ± SD		
	24 hours	7 days	15 days
BNC/C	7.35 ± 0.22 ^a,A^	8.08 ± 0.04 ^ab,A^	8.63 ± 0.2 ^b,A^
BNC/CHX	7.04 ± 0.22 ^a,A^	8.08 ± 0.03 ^b,A^	8.49 ± 0.23 ^b,A^
BNC/CLIN	6.05 ± 0.21 ^a,B^	7.85 ± 0.04 ^a,B^	8.33 ± 0.24 ^b,A^
BNC/AMOX	4.04 ± 0.18 ^a,C^	7.9 ± 0.03 ^a,B^	8.41 ± 0.25 ^b,A^

Different lowercase letters in the same row and different uppercase letters in the same column indicate a significant difference. Two-way ANOVA and Bonferroni’s post-hoc multiple comparisons (α=5%).

### SEM analysis

**Figure 1:**
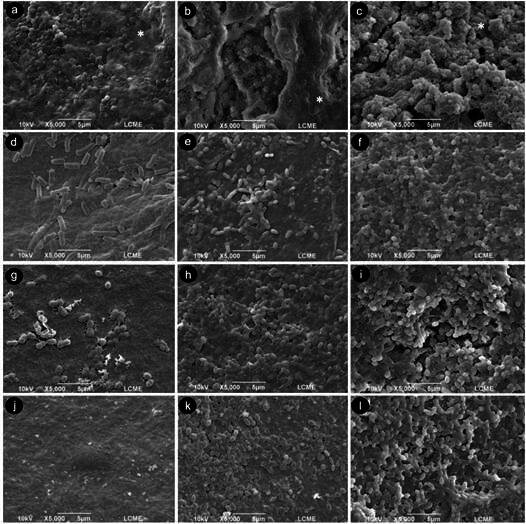
Representative SEM images of the different BNC scaffolds after the experimental periods of incubation (24 hours, 7 and 15 days - left to right). BNC/C (A-C) Regular, homogeneous, and denser multispecies biofilm, with the extracellular matrix layer (*) covering the entire nanofiber network surface, regardless of the period of analysis. BNC/CHX (D and E) Isolated bacterial cells and small bacterial clusters were distributed on the surface of the nanofiber network in the initial periods of analysis. (F) Dense and homogeneous multispecies biofilm covering the nanofiber network surface in the 15-day period. BNC/CLI (G) Prevalence of rod-shaped microorganisms, characteristic of *A. naeslundii,* in the 24-hour period. (H and I) Note the presence of cocci-type microorganisms adhered to the scaffolds incorporated with clindamycin over the course of the experimental periods. BNC/AMOX (J) No microorganisms in the 24-hour period. (K and L) Note a more complex formation, with greater aggregation of microorganisms over the course of the experimental periods. Magnification of 5,000×.

The SEM analysis conducted after incubation periods of 24 hours, 7, and 15 days revealed adherent microorganisms on the BNC scaffolds, irrespective of the experimental condition (Figure 1).

During the initial period of analysis (24 hours), fewer microorganisms were observed in the experimental groups than in the negative control group (oxidized BNC scaffolds without antimicrobial agents). In addition to this numerical difference, qualitative variations in the early colonization profile were also evident. BNC/C exhibited a more established and compact biofilm, characterized by a dense extracellular matrix covering the nanofiber network and abundant bacterial colonization with a heterogeneous mixture of *cocci* and short rods (Figure 1A). In contrast, the experimental groups displayed less mature colonization. BNC/CLI showed sparse bacterial adhesion composed predominantly of rod-shaped microorganisms compatible with A. naeslundii (Figure 1G), while BNC/CHX and BNC/CLI exhibited limited early matrix formation and clusters of dispersed cocci. Notably, no microorganisms were observed on BNC/AMOX at 24 hours (Figure 1J), indicating potent initial inhibition.

By day 7, all groups showed more continuous microbial coverage. BNC/C showed a thickened, well-organized biofilm layer with evident extracellular polymeric substance (EPS) structure, whereas BNC/CHX, BNC/CLI, and BNC/AMOX scaffolds displayed intermediate biofilm maturation, with less dense EPS and more defined bacterial morphotypes. At 15 days, a mature and homogeneous multispecies biofilm was observed across all experimental conditions, fully covering the scaffold surface. While bacterial density appeared comparable among groups at this stage, the structural organization of the EPS matrix was slightly looser in the antimicrobial-containing scaffolds (Figures 1B-1L), consistent with the early inhibitory effects observed in the CFU analysis.

### CLSM analysis

**Figure 2:**
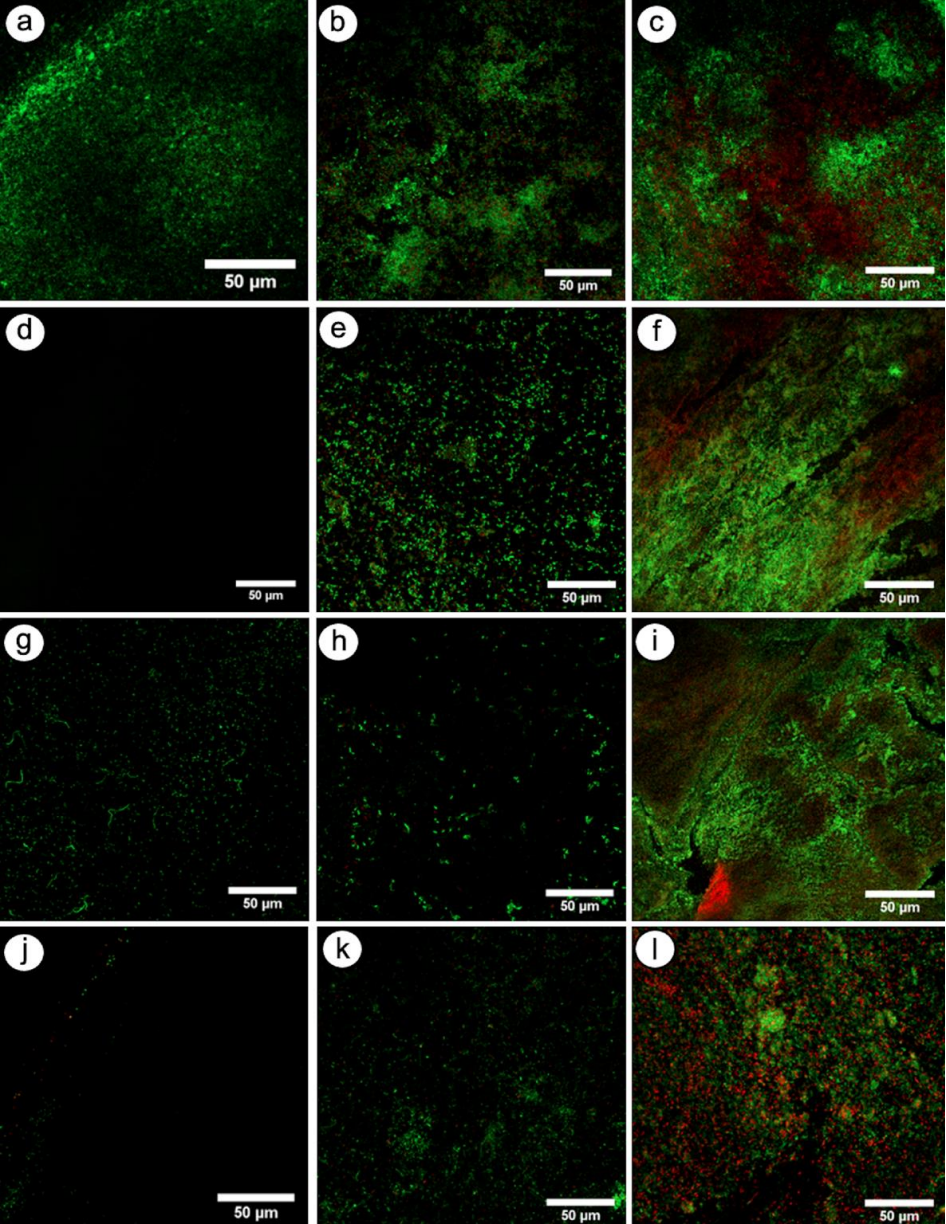
Representative CLSM images of the different BNC scaffolds after the experimental periods of incubation (24 hours, 7, and 15 days - left to right). Green colour (SYTO 9) indicates viable microbial cells, and red color (propidium iodide) indicates non-viable cells. BNC/C **(A-C)** shows substantial proportions of viable microorganisms adhered to the nanofiber network surface, particularly at the initial experimental periods, serving as the reference for comparison with the experimental groups. Compared with BNC/C, BNC/CHX **(D-F)**, BNC/CLI **(G-I)**, and BNC/AMOX **(J-L)** showed reduced viable microorganism counts at 24 hours and 7 days. At the 15-day period, a greater proportion of non-viable microorganisms (red) adhered to the BNC scaffolds was observed across all groups, regardless of the experimental condition. All bars represent 50 µm.

The CLSM analysis demonstrated the presence of both dead and live microorganisms adhered to all BNC scaffolds, irrespective of the experimental condition (Figure 2).

BNC/C exhibited uniform biofilm growth with a substantial amount of viable microorganisms (green) adhered to the nanofiber network surface, particularly during the early periods of analysis (24 hours and 7 days) (Figure 2A and Figure 2B). Compared with the negative control group, BNC/CHX (Figure 2D and Figure 2E), BNC/CLI (Figure 2G and Figure 2H), and BNC/AMOX (Figure 2J and Figure 2K) showed reduced bacterial proliferation at 24 hours and 7 days. By the 15-day period, no noticeable differences among the groups were observed. Additionally, in this final period, a greater proportion of non-viable microorganisms (red) was observed on the scaffolds across all experimental conditions.

## Discussion

BNC scaffolds incorporated with different antimicrobial agents may be a viable alternative for controlling infection within the root canal system, especially for immature necrotic teeth ^(^ ^15^ ^,^ ^20^. The synthesis of calcium carbonate microparticles within the fibers of BNC enables it to serve as a structure capable of incorporating an antimicrobial agent and releasing it slowly and specifically ^(^ ^10^.

In the present study, the antibiofilm activity of the BNC scaffolds incorporated with 0.12% chlorhexidine digluconate and different antibiotics (1% clindamycin and 1% amoxicillin) was assessed at different time points of incubation. Based on our results, the first null hypothesis was partially rejected. At the 24-hour incubation period, BNC/AMOX and BNC/CLI demonstrated significantly lower CFU counts compared to BNC/CHX and BNC/C, indicating superior antibiofilm activity in the short term. BNC/AMOX was particularly effective, exhibiting the lowest number of viable microorganisms at this period. However, such differences diminished over time, as all groups showed similar CFU values at 15 days, suggesting that the antimicrobial efficacy of BNC/AMOX and BNC/CLI was not sustained in the long term. Thus, while BNC/AMOX and BNC/CLI outperformed BNC/CHX and BNC/C initially, the overall performance of the scaffolds converged after extended incubation.

The second null hypothesis was also partially rejected. BNC/AMOX and BNC/CLI exhibited a significant increase in bacterial adherence from 24 hours to 15 days, evidencing a progressive loss of antimicrobial efficacy over time. Similarly, BNC/CHX showed lower bacterial viability at 24 hours, with a significant increase observed at 7 days that persisted at 15 days. In contrast, BNC/C maintained relatively stable CFU values across the first two incubation periods, with a significant increase detected only at 15 days. These findings indicate that the antimicrobial performance of the scaffolds is time-dependent, with the most pronounced activity observed at the early incubation stage, followed by a gradual decline in efficacy.

Overall, BNC/AMOX exhibited superior antibiofilm effect in the initial periods when compared to the other groups. Penicillins, in general, are the most commonly used antimicrobial agents for odontogenic infections due to their historical efficacy, low toxicity, and low cost-effectiveness ^(^ ^21^. They act by inhibiting the activity of bacterial proteins involved in the synthesis of peptidoglycan cell walls in both gram-positive and gram-negative bacteria ^(^ ^22^. Amoxicillin, in particular, is a broad-spectrum antibiotic ^(^ ^21^ with antimicrobial properties against various microorganisms, including *E. faecalis*, which is highly associated with endodontic infections ^(^ ^23^. Bacteria associated with endodontic abscesses often exhibit high susceptibility to this medication when administered systemically ^(^ ^24^. Although a sustained medication release has been demonstrated when incorporated into collagen matrices ^(^ ^25^, there is a lack of scientific studies incorporating amoxicillin into scaffolds in REPs. Therefore, the findings of our study bridged an important gap in the literature.

BNC scaffolds incorporated with 1% clindamycin also exhibited lower bacterial adhesion in the 24-hour and 7-day periods than BNC/CHX and BNC/C. However, its efficacy was inferior in comparison with BNC/AMOX in the first 24 hours of incubation. Clindamycin is the first-choice medication for treating patients allergic to penicillin ^(^ ^26^. It is a bacteriostatic lincosamide with antimicrobial efficacy against a broad spectrum of bacteria found in endodontic infections (gram-positive aerobes and most anaerobic bacteria) ^(^ ^27^ ^,^ ^28^. A previous study that also incorporated clindamycin into BNC scaffolds demonstrated similar antimicrobial and antibiofilm potential within a 24-hour period against a multispecies biofilm ^(^ ^15^. However, when this antibiotic was incorporated into other types of scaffolds, it showed species-dependent action ^(^ ^27^. Despite being able to inhibit the growth of various species, such as *E. faecalis*, *A. naeslundii*, *A. actinomycetemcomitans*, and *F. nucleatum* ^(^ ^27^, 1% clindamycin has demonstrated a greater efficacy against *A. naeslundii*, a rod-shaped microorganism ^(^ ^27^. In our study, the multispecies biofilm was composed of three facultative anaerobic bacteria commonly found in cases of pulp necrosis in teeth with incomplete root formation, including *A. naeslundii*. In the SEM analysis, a predominance of cocci-type microorganisms adhered to the nanofiber network was observed in the initial 24 hours, corroborating the efficacy of 1% clindamycin against *A. naeslundii* ^(^ ^27^.

In the current research, BNC/CHX exhibited inferior results compared to the other experimental scaffolds. Recent studies have also demonstrated similar findings, where BNC scaffolds incorporated with 0.12% chlorhexidine digluconate exerted a satisfactory antimicrobial and antibiofilm activity despite showing adverse cytotoxic effects ^(^ ^15^. Chlorhexidine digluconate has been recommended both as an irrigating solution ^(^ ^29^ ^,^ ^30^ and intracanal dressing due to its antimicrobial activity ^(^ ^30^ ^,^ ^31^. It is a broad-spectrum antimicrobial agent against gram-positive and gram-negative bacteria and yeasts, including *E. faecalis* ^(^ ^29^. Its action occurs through negatively charged cationic ions that are attracted to the inner membrane of bacteria, exerting a bactericidal effect ^(^ ^32^. However, its efficacy is concentration-dependent ^(^ ^29^. At concentrations of 0.1% and 0.2%, it is bacteriostatic; at higher concentrations, such as 2%, it becomes bactericidal ^(^ ^33^. Therefore, the concentration of chlorhexidine digluconate used in this study (0.12 mg/mL) may have been lower than necessary to achieve the expected bactericidal effect.

A proper concentration of substances used in root canal disinfection should be sufficient to achieve a bactericidal effect ^(^ ^33^ ^,^ ^34^ ^,^ ^35^. However, concerning REPs, a concentration compatible with the survival of stem cells must be taken into consideration ^(^ ^36^. For this reason, the concentration of substances commonly used in the chemical disinfection of the root canal system during REPs, such as TAP, is low, ranging from 0.1 to 1.0 mg/mL ^(^ ^36^. In our study, the lower concentration of the chlorhexidine digluconate (0.12 mg/mL) and the antibiotics (0.1mg/mL) incorporated into the BNC scaffolds was insufficient to eradicate microorganisms and provide a long-term antibiofilm effect.

The expected prolonged antimicrobial action of the BNC scaffolds was not evident, as significant biofilm growth was observed in all groups, especially in the 15-day period. This finding may be related to the drug release being inversely proportional to the concentration incorporated into the nanofiber, releasing a smaller percentage of the drug as its load increases ^(^ ^10^. Additionally, drug release kinetics may be altered by variations in the local microenvironment pH, with higher release observed at lower pH ^(^ ^20^. The difference in the molecular weight of antibiotics was also suggested by Bottino et al. ^(^ ^20^ as a plausible reason for differences in drug release.

Regarding the methodological aspects of this study, the synthesis, characterization, and purification protocols of the scaffolds, established in BNC-related literature ^(^ ^15^ ^,^ ^20^ ^,^ ^37^, ensured consistent formation of mechanically stable nanofibrous structures with reproducible morphology and physicochemical characteristics. This intrinsic characterization of the material, based on validated and standardized methods ^(^ ^15^ ^,^ ^20^ ^,^ ^37^, guarantees the absence of microbial residues that could interfere with subsequent assays.

The clinical relevance of this study lies in the ongoing search for effective intracanal disinfection strategies for REPs, particularly in immature teeth with persistent infection. Therefore, further investigation into the effectiveness of the controlled drug-release system incorporated into BNC scaffolds is warranted ^(^ ^15^ ^,^ ^20^.

Additionally, to establish an ideal disinfection protocol in REPs, studies evaluating different types of microorganisms and determining the optimal concentration of each medication capable of promoting prolonged antimicrobial effects without compromising stem cell viability are necessary ^(^ ^38^. Importantly, future research should also assess the antimicrobial efficacy of BNC scaffolds against mature, well-established biofilms, as this condition more closely reflects the clinical reality of persistent infections in REPs and is one of the most challenging scenarios for achieving predictable disinfection.

## Conclusion

Based on our findings, BNC scaffolds containing 1% amoxicillin and 1% clindamycin demonstrated immediate antimicrobial activity against a multispecies biofilm. This effect was not observed at late incubation periods. Conversely, the suggested slow-release drug system was ineffective, as over the final experimental period, the BNC scaffolds allowed the formation of a multispecies biofilm.

## Data Availability

The research data are available upon request
